# Cost Effectiveness Analysis of Disease-Modifying Antirheumatic Drugs in Rheumatoid Arthritis. A Systematic Review Literature

**DOI:** 10.1155/2011/845496

**Published:** 2011-11-22

**Authors:** Maurizio Benucci, Gianantonio Saviola, Mariangela Manfredi, Piercarlo Sarzi-Puttini, Fabiola Atzeni

**Affiliations:** ^1^Rheumatology Unit Department of Internal Medicine, Hospital S. Giovanni di Dio, Azienda Sanitaria di Firenze, 50143 Florence, Italy; ^2^Rheumatology and Rehabilitation Unit, Salvatore Maugeri Foundation IRCCS, 46042 Mantua, Italy; ^3^Immunology and Allergology Laboratory Unit, Hospital S. Giovanni di Dio, Azienda Sanitaria di Firenze, 50143 Florence, Italy; ^4^Rheumatology Unit, Sacco University Hospital, 20127 Milan, Italy; ^5^Experimental Medicine and Rheumatology, William Harvey Research Institute, Queen Mary University of London, London E14DG, UK

## Abstract

The cost effectiveness of treatments that have changed the “natural history” of a chronic progressive disease needs to be evaluated over the long term. Disease-modifying antirheumatic drugs (DMARDs) are the standard treatment of rheumatoid arthritis (RA) and should be started as early as possible. A number of studies have shown that they are effective in improving disease activity and function, and in joint damage. Our review was focused on revision and critical evaluation of the studies including the literature on cost effectiveness of DMARDs (cyclosporine A, sulphasalazine, leflunomide, and methotrexate). The European League Against Rheumatism (EULAR) recommendations showed that traditional DMARDs are cost effective at the time of disease onset. They are less expensive than biological DMARDs and can be useful in controlling disease activity in early RA.

## 1. Introduction

Rheumatoid Arthritis(RA) affects about 272,000 Italian patients. Population data have showm that the prevalence of RA in Italy is 0.46% [1]. In 2002, it was estimated that the socioeconomic cost of RA in Italy was 1600 million euros (1210 million for indirect social costs and 380 million for direct medical costs). The calculated average annual cost for each patient varies significantly depending on disease severity and correlates with the Health Assessment Questionnaire Disability Index (HAQ-DI) and American College of Rheumatology (ACR) Steinbrocker criteria, from *€*3,718.3 in class I to *€*22,946 in class IV [2].

The economic impact of RA is substantial in terms of direct, indirect, and intangible costs. Direct costs are those related to RA, such as medications, clinic visits, hospitalisations, aids, devices, and transportation; indirect costs include the costs incurred by society as a result of lost productivity and reduced patient and family incomes: intangible costs are those arising from the reduced quality of life and premature mortality. As RA is a chronic inflammatory joint disease that mainly affects people of working age, its costs are considerable [3].

The cost effectiveness of treatments that can change the “natural history” of a chronic progressive disease has to be evaluated over the long term using models that allow varying the assumptions of treatment continuation and long-term effects and assess the uncertainty concerning results, including those from the clinical trials. There are many approaches to modelling costs and outcomes and assessing cost effectiveness, including the use of disease models based on epidemiological data in which costs and the quality of life (utility) are related to the degree of disease severity and progression [4]. In models of RA, disease severity is based on functional status as measured by the HAQ, and outcomes are expressed as quality-adjusted life years (QALYs) [5]. The cost effectiveness estimates are based on the concept that treatment will prevent progression to the next level(s) of disease severity or will take longer time to progress, thus avoiding or delaying the high costs and low utility levels associated with more severe disease [6].

Recent Italian population data have shown that the prevalence of RA has increased to 0.49% [7], in line with the findings of epidemiological studies carried out in the United State (US) [8]. The incidence of new cases is increasing [9], but it has not been followed by an increase in the use of biological therapy, which was prescribed for only 6.3% of RA patients in 2009 [10]. 

The purpose of this paper is to review and critically assess the existing literature on the cost effectiveness of traditional DMARDs.

## 2. Cost Effectiveness of DMARDs in RA

When considering RA drug therapy, a number of factors seem to predict the cost-effectiveness analysis. First of all, disability is the major determinant of cost, and so drugs that slow joint destruction and delay the onset of disability may therefore be cost effective. Secondly, the costs related to adverse events form a major portion of the real drug costs; therefore, a drug with a good safety profile will probably be more cost effective than one that shows similar efficacy but has more side effects. Finally, it must be remembered that RA drug therapy must be efficacious and safe over the long term.

DMARDs are the standard treatment of RA and should be started as early as possible. A number of studies have shown that they are effective in improving disease activity and function, and delaying joint damage. However, there are not many economic analyses of DMARD treatment in RA [11].

## 3. Methods

To collect and review the evidence, we performed a systematic literature review (SLR) aiming at economic evaluations of RA treatment. The search used Medline, Embase, and Cochrane databases to identify publications concerning economic aspects of adult RA, published in English from the year of database inception until June 2011. We evaluated the main cost effectiveness studies of the DMARDs currently used in RA using the search words DMARDs (cyclosporine A, sulphasalazine, leflunomide, and methotrexate), cost effectiveness analysis, and rheumatoid arthritis.

The selected papers included randomised controlled trials (RCTs) and observational studies (prospective and retrospective cohort and case-control studies).

In addition, we hand-searched abstracts presented at the 2007 and 2008 ACR and EULAR conferences. 

Where possible, we collected cost effectiveness results as an incremental cost-effectiveness ratio (ICER), the most frequently reported outcome in cost-effectiveness analyses (CEAs) reflecting the additional cost per QALY gained by a particular treatment. Although the exact societal willingness-to-pay (WTP) threshold remains controversial, based on ICERs of other commonly accepted medical interventions, we defined ICERs below US $50.000–100.000 or *£*30.000 or *€*50.000 per QALY, as being cost effective [12].

## 4. Results

We identify in PubMed database using the following words DMARDs (cyclosporine A, sulphasalazine, leflunomide, and methotrexate), cost effectiveness analysis, and rheumatoid arthritis a total of 86 studies.

Out of 86 studies, 33 were excluded due to the comparison between cost-effectiveness analysis of DMARDs and anti TNF alpha agents (6 studies on etanercept, 3 on adalimumab, 7 on infliximab, and 1 on certolizumab pegol). 

Furthermore, we excluded a total of 6 studies because they were focused on comparison between cost-effectiveness analysis of DMARDs and other biological agents (2 studies on anakinra, 2 on abatacept, 1 on tocilizumab, and another one on rituximab).

On the basis of selection, we evaluated one cost-effectiveness meta-analysis for cyclosporine A (CyA), one paper of step-down therapy with sulphasalazine (SSZ), three different papers that compared leflunomide (LNF) alone or against other DMARDs (SSZ and methotrexate), and five papers that evaluated methotrexate in monotherapy, in combination with hydroxychloroquine (HCQ), SSZ, and against LNF. Furthermore, we considered the three recent papers that compared the use of DMARDs in particular methotrexate (MTX) in early or long-standing RA evaluated with biological anti-TNF alpha inhibitors (Table 1).

### 4.1. Cyclosporine A

The cost effectiveness of CyA in the treatment of RA has been investigated in a meta-analysis of five randomised, controlled, and parallel-group clinical trials that used a fixed effects model to calculate treatment effects and incremental economic analyses from the perspectives of society and the Ontario Ministry of Health (MoH). Comparisons were made with placebo, and there were two head-to-head comparisons. The total treatment cost for a typical patient was calculated using a modified intention-to-treat approach modelled over one year. CyA led to a ≥25% improvement in tender joint counts in 35% of the patients against 17% of the patients receiving placebo; there was no significant difference between CyA and azathioprine (AZA) or D-penicillamina (D-Pen). From the perspective of the Ontario MoH, the annual incremental cost of achieving the same level of improvement was $1,473 between CyA and AZA, and $1,618 between CyA and D-Pen; the annual incremental cost effectiveness ratio per patient improved between CyA and placebo was $11,547. From the point of view of society, the incremental cost of CyA was $2,886 over AZA, and $3,731 over D-Pen, and the annual incremental cost effectiveness ratio against placebo was $20,698 [13].

### 4.2. Sulphasalazine

The cost effectiveness and cost utility of early intervention in RA patients have been investigated by comparing combined step-down prednisolone, MTX, and SSZ (76 patients) with SSZ alone (78 patients). The mean total cost per patient in the first 56 weeks of follow-up was $5519 for the combined treatment and $6511 for treatment with SSZ alone (*P* = 0.37). Outpatient, inpatient and nonhealth care each contributed about one-third to the total cost. The combined treatment group appeared to generate savings in terms of the duration of hospitalisation because of RA, nonprotocol drugs, and the costs of home help although protocol drugs and monitoring were slightly more expensive. Clinical, radiographic, and functional outcomes significantly favoured combined treatment at week 28 (and radiographic outcome also at week 56). The utility scores also favoured combined treatment [14].

### 4.3. Leflunomide

A DMARD sequence including LFN has been compared with one excluding it using a 5-year simulation model in which the patients cycle through different treatment regimens. The data were obtained by means of a systematic review of the literature (drug withdrawal rates, the number and type of adverse events, and ACR 20% responder status) and separately surveyed (the choice of DMARD sequence, the management of adverse events). The costs of adverse event management were calculated using the Ontario Schedule of Benefits, and monitoring of costs was calculated according to official Canadian product monograph recommendations. Utilities (as measured by the standard gamble (SG) and rating scale (RS) techniques) were obtained from 482 patients who participated in a 1-year randomised controlled trial comparing LFN, MTX, and placebo. Adding LFN to a conventional DMARD strategy increased 5-year management costs by $1,231, which leads to a cost-effectiveness ratio of $13,096 per additional year of response to treatment, and cost-utility ratios of $54,229 (RS) and $71,988 (SG) per QALY gained [15]. The annualised total RA- or drug-related costs for LNF, MTX, and placebo were, respectively, $1761, $1280, and $1324, and the medical care costs were, respectively, $753, $620, and $167 (*Canadian dollars*) (all costs excluding drug acquisition and monitoring costs). The estimated annual drug acquisition/routine monitoring costs were 3853/483 Canadian dollars for LNF and 258/599 Canadian dollars for methotrexate. The differences between the overall costs (excluding drug acquisition and monitoring costs) and medical care costs were not statistically significant. The cost of treating patients with LNF was significantly higher than that of treating them with methotrexate when drug acquisition and monitoring costs were included (*P* < 0.0001) [16]. 

A Markov model has been constructed using the health states defined by the HAQ score. This model is based on a cohort of patients with recently diagnosed definite RA who were followed for up to 15 years by nine rheumatology clinics in the United Kingdom (UK). The treatment effect was calculated on the basis of clinical trials comparing LFN and MTX (one international and one US trial) or sulphasalazine (one international trial). Using the US trial, LFN had slightly lower costs and better effects than MTX (*£*44,017 and 4,307 QALYs versus *£*44,988 and 4.158 QALYs), but this was reversed when using international trial (*£*36,351 and 4,372 QALYs versus *£*34,070 and 4.487 QALYs). In comparison with sulphasalazine, the cost of using LEF was slightly lower, and there was an increase in QALYs (*£*35,855 and 3.896 QALYs versus *£*36,731 and 3.721 QALYs). The reason for the different results when using the two trials comparing LFN with MTX was possibly that the MTX patients in the US trial were also treated with folic acid, whereas folate supplementation was not mandatory in the international trial; this may have reduced the effectiveness of MTX. In comparison with sulphasalazine, the use of LFN appears to be cost effective in the UK [17].

### 4.4. Methotrexate

A cost effectiveness analysis has been made using a decision model with a time horizon of six months and comparing six treatment options for patients with MTX-resistant RA: (1) etanercept + MTX, (2) etanercept monotherapy, (3) CyA + MTX, (4) triple therapy (hydroxychloroquine, sulphasalazine, and MTX), (5) the continuation of MTX monotherapy, and (6) no second-line agent. Two measures of effectiveness based on published clinical trial data were used: the ACR 20% response criteria (ACR 20), and a weighted average of the proportion of patients achieving ACR 70, ACR 50, and ACR 20 responses (ACR 70 weighted response (ACR 70WR)). The results showed that MTX therapy for MTX-naive RA patients cost $1,100 per ACR 20 outcome and $1,500 per ACR 70WR, in comparison with no second-line agent. The least expensive option was triple therapy, which cost 1.3 times more per patient with an ACR 20 outcome ($1,500/ACR 20) and 2.1 times more per ACR 70WR ($3,100/ACR 70WR) than MTX therapy in MTX-naive RA patients. The analysis indicates that if 15 mg/week MTX is cost effective for achieving ACR 20 or ACR 70WR in MTX-naive RA patients over a 6-month period, it is likely that the triple therapy is also cost effective in patients with MTX-resistant RA [18]. 

The same authors in MTX-naïve RA patients showed that MTX is cost effective (cost saving versus the no-second line agent option) for achieving ACR 20 or ACR 70WR over a 6-month period. However, based on available data, the relative cost effectiveness of SSZ and MTX cannot be determined with reasonable certainty although SSZ therapy seems to be cost effective as MTX (cost saving) for achieving ACR outcomes over a 6-month period [19].

Data concerning disease activity, functional status, and society costs have been collected from a 1-year cohort of 152 patients with RA receiving at least one DMARD for six months. Treatment with MTX + antimalarials (AMs) would save as much as US $834 per 1 unit (U) of HAQ improvement per year in comparison with AMs alone. Other DMARD options with MTX or LFN were more expensive than AMs but were more efficacious and considered cost effective. Treatment with these DMARDs would incur additional costs of US $625–2,061 per 1 U of HAQ improvement per year. DMARD options without MTX were not cost effective [20]. 

An analysis from the perspective of society has been made in Germany using a modelling approach based on the secondary analysis of existing data and on data obtained from a sample of 583 patients included in the German rheumatological database of 1998. Functional capacity was defined on the basis of Hannover Functional Ability Questionnaire (HFAQ) scores, and the costs were calculated on the basis of resource use and the patients' working capacity. The average total direct costs (1998–2001 values) per patient per year for continuous treatment with the selected DMARDs, including purchase costs and the monitoring and treatment of adverse drug reactions (ADRs), were highest for intramuscular gold (sodium aurothiomalate) (*€*2106; *€*1 H $US 0.91, the average of the period 2000-2001) followed by LFN (*€*2010), AZA (*€*1878), sulphasalazine (*€*1190), oral MTX (*€*708), and lowest for the AMs: chloroquine/hydroxychloroquine (*€*684) [21]. 

The effect of indirect costs for patients with early RA in the COBRA trial (Combinatie therapie Bij Reumatoide Artritis) on the cost effectiveness of the treatment with prednisolone, MTX, and SSZ (the COBRA combination) or with SSZ alone has been shown that, in the first 28 weeks, indirect costs per patient were US$2,578 for COBRA and US$3,638 for SSZ (*P* = 0.09); the total costs were, respectively, $5,931 and $7,853 (*P* < 0.05) [22].

A randomized-double blind-placebo-controlled trial on cost effectiveness of folic or folinic acid supplementation in RA patients treated with MTX using EuroQol questionnaire to assess the QALY showed no significant differences among patients taking folic or acid folic supplementation or placebo over a 6-month followup [23].

## 5. Discussion

Given the increase in health-related costs and limited health care resources, assessing the cost effectiveness of medical procedures is gaining considerable importance in the field of rheumatology; particularly, as the annual therapy costs of using TNF-blocking agents such as etanercept and infliximab (*€*17,000–21,000) are much higher than those of using DMARDs in the treatment of RA [24]. TNF inhibitors are currently considered both effective and cost effective in patients with active RA, especially in patients who have not fully responded to MTX, but the data suggest that, in routine clinical practice, they provide only modest incremental benefits over the best conventional therapy. If confirmed, these observational findings suggest that the economic issue underpinning the widespread use of TNF inhibitors in established RA is unsustainable [25].

In a previous real-life postmarket observational study, we demonstrated that etanercept plus MTX was cost effective with a QALY lower than the acceptable threshold of *€*50,000, and MTX was more cost effective than LFN [26]. The efficacy of LFN is comparable with that of SSZ and MTX, but LFN is more expensive than most DMARDs, and costs about *£*400 per year more than SSZ [27]. 

Three management strategies have been compared: a symptomatic or “pyramid” strategy starting with nonsteroidal anti-inflammatory drugs, patient education, pain management, and low-dose glucocorticoids, then switching nonresponders to DMARDs after one year; early DMARD therapy with MTX; early therapy with biological agents and MTX. When the cost of very early intervention was factored in, the cost effectiveness ratio of the early DMARD strategy was $4849 per QALY (95% CI: $0–16,354 per QALY) in comparison with the pyramid strategy, whereas the benefits gained using the early biological strategy came at a substantially increased cost. Very early intervention with conventional DMARDs is cost effective, whereas the cost effectiveness of very early intervention with biological agents remains uncertain [28]. 

A systematic review of 18 papers has shown that biological agent/DMARDs sequences are cost effective in DMARD-naïve patients for the threshold Canada dollars (CAD) 100,000/QALY. In the patients failing on combined MTX therapy or sequentially administered DMARDs, cost-effectiveness ratios (ICERs) were well above CAD 50,000/QALY, and 40% were below CAD 100,000/QALY. The most cost effective approach seems to be DMARD treatment early in the course of RA, then moving to a DMARD sequence in the case of a nonresponse, and finally adding a biological drug if the nonresponse continues [29]. 

A review of the cost effectiveness of RA treatments and the clinical recommendations of EULAR has shown that DMARDs are cost effective at disease onset, that is, that the merits of treatment outweigh its costs. If DMARDs fail, therapeutic escalation with TNF inhibitors is cost effective when standard-dosing regimens are used. If TNF inhibitors fail, rituximab or abatacept is cost effective. There is little economic evidence for switching TNF inhibitors [30]. MTX and biological agents have been found to be similarly effective as measured by ACR and EULAR response criteria, including clinical remission although folic acid supplementation may have reduced the efficacy of MTX by interfering with its mechanism of action. 

Nonetheless, all of the trials have confirmed the surprisingly good performance of MTX in comparison with biological agents [31].

A validated Markov model was used to evaluate the cost effectiveness of all three strategies, while a daily practice data from two different cohorts was used to determine the effectiveness of the strategies. Patients treated according to these strategies were matched for baseline DAS-28. The expected costs, the QALYs, and the incremental cost per QALY gained over a 5-year time were calculated using a Monte Carlo simulation, from health-care and societal perspectives. Starting with following combination therapy (MTX plus LFN or plus anti-TNF) is more expensive than starting with MTX in monotherapy. Furthermore, starting with anti-TNF therapy is far more expensive than starting with MTX in monotherapy [32].

Conventional DMARDs are much less expensive than biological DMARDs and, in many cases, can lead to similar control of disease activity, particularly in early RA. The goal for all patients should be to obtain remission or at least a low level of disease activity using the most cost-effective therapy possible.

## Figures and Tables

**Table 1:** Studies on cost effectiveness analysis of DMARDs in rheumatoid arthritis.

Authors and years of publication	Type of the drugs	Countries	Overall time	Evaluation's model	Outcomes	Cost/Effective/Ratio
Anis et al. 1996 [13]	CyA versus AZA versus D-Pen	Canada	12 months	ITT	ICER	20,698$
Verhoeven et al. 1998 [14]	SSZ versus COMBO	Netherland	56 weeks		QALY	6511 versus 5519$
Maetzel et al. 2002 [15, 16]	LNF	Canada	12 months	ACR 20	QALY	54229$
Maetzel et al. 2002 [15, 16]	LNF versus MTX	Canada	12 months	ACR 20	QALY	3853$
Kobelt et al. 2002 [4, 17]	LNF versus MTX versus SSZ	UK	15 years	Markov	QALY	35855 versus 44988 versus 37731*£*
Bruns et al. 2000 [18]	MTX versus ETN versus MTX + ETN versus MTX + CyA versus HCQ + MTX + SSZ	USA	6 months	ACR 20 ACR 70WN	Tree model	1100 versus 1500$
Choi et al. 2002 [19]	MTX versus ETN versus LNF versus SSZ	USA	6 months	ACR 20 ACR 70WN	Tree model	900 versus 1500$
Osiri et al. 2007 [20]	MTX versus DMARDs	Thailand	1 year	HAQ	ICER	834 versus 2061$
Schädlich et al. 2005 [21]	MTX versus LFN	Germany	1 year	HFAQ	Tree	708 versus 2010*€*
Korthals-De Bos et al. 2004 [22]	COBRA versus SSZ	Netherland	28 weeks	HAQ	QALY	2578 versus 3638$
Hartman et al. 2004 [23]	MTX versus MTX + folic acid	Netherland	48 weeks	EuroQol	QALY	1398 versus 1776$
Schipper et al. 2011 [32]	MTX versus MTX + LNF versus MTX + TNF	Netherland	5 years	Markov	QALY	16620 versus 17574$

CyA: cyclosporine A; HCQ: hydroxychloroquine; TNF: tumour necrosis factor; LNF: leflunomide; MTX: methotrexate; ETN: etanercept; SSZ: sulphasalazine; DMARDs: disease modifying antirheumatic drugs; ACR: American College of Rheumatology; HAQ: health assessment questionnaire; ITT: intention to treat; QALY: quality-adjusted life years; ICER: incremental cost-effectiveness ratio HFAQ: Hannover Functional Ability Questionnaire; UK: United Kingdom; USA: Unite States of America.
